# Hepatoprotective effects of *Tagetes lucida* root extract in carbon tetrachloride-induced hepatotoxicity in Wistar albino rats through amelioration of oxidative stress

**DOI:** 10.1080/13880209.2021.1949024

**Published:** 2021-08-04

**Authors:** Samah Ali El-Newary, Rasha Fouad Ismail, Nermeen Mohammed Shaffie, Saber Fayez Hendawy, Elsayed Omer, Mahgoub Mohammed Ahmed, Wael M. ELsayed

**Affiliations:** a Medicinal and Aromatic Plants Research Department, National Research Centre, Giza, Egypt; b Pathology Department, Medical Researches Division, National Research Centre, Giza, Egypt; c Molecular Drug Evaluation Department, National Organization for Drug Control and Research (NODCAR), Giza, Egypt; d Chemistry of Medicinal Plants Department, Pharmaceutical and Drug Industries Research Division, National Research Centre, Giza, Egypt

**Keywords:** Anti-inflammatory effect, CYP2E1 inhibitor, antioxidant characteristics

## Abstract

**Context:**

The roots of *Tagetes lucida* Cav. (Asteraceae) have antioxidant and antimicrobial properties.

**Objective:**

This study aimed to examine the hepatoprotective effects of *T. lucida* roots ethanol extract (TLRE) using carbon tetrachloride (CCl_4_)-induced hepatotoxicity in rats.

**Materials and methods:**

The active ingredients of TLRE were identified by high-performance liquid chromatography, infra-red spectrum, and mass spectrometric procedures. Ninety rats were distributed into four main groups: positive, therapeutic, protective, and negative group. The therapeutic group was implemented using CCl_4_ (a single dose of 2 mL/kg) before TLRE or silymarin administration. Meanwhile, the protective group was implemented by administering CCl_4_ (a single dose of 2 mL/kg) after force-feeding TLRE or silymarin. Each therapeutic and protective group was divided into three subgroups: force-fed with saline, TLRE (500 mg/kg), and silymarin (25 mg/kg). The positive group was split into two subgroups that were force-fed TLRE and silymarin. Positive, therapeutic, and protective groups were compared to the negative group (untreated rats). CCl_4,_ TLRE, and silymarin were orally administrated using a gastric tube.

**Results:**

In the therapeutic and protective groups, TLRE significantly reduced liver enzymes, i.e., aspartate aminotransferase (12.47 and 6.29%), alanine aminotransferase (30.48 and 11.39%), alkaline phosphatase (17.28 and 15.90%), and cytochrome P450-2E1 (39.04 and 48.24%), and tumour necrosis factor-α (53.72 and 53.72%) in comparison with CCl_4_-induced hepatotoxicity controls.

**Conclusions:**

TLRE has a potent hepatoprotective effect with a good safety margin. After a repeated study on another type of small experimental animal, their offspring, and an experiment with a large animal, this study may lead to clinical trials.

## Introduction

The liver is the body’s most vital organ. It has many functions including, nutrients metabolism, waste metabolites excretion, and xenobiotics detoxification. Several toxic substances such as carbon tetrachloride (CCl_4_) and thioacetamide can infect hepatocytes. These toxicants induce lipid peroxidation and oxidative stress. The industrial progress that facilitates the modern lifestyle has poisoned the environment through poor control of industrial waste disposal. One toxicant in such waste is CCl_4_, emitted from chemical industries and accumulates in the atmosphere. The CCl_4_-induced hepatotoxicity model is used to study the hepatoprotective effects of drugs and plant extracts. CCl_4_-induced hepatotoxicity is caused by **a)** the promotion of lipid peroxidation, **b)** inhibition of antioxidant enzymes, and **c)** induction of free radical production (Kumar et al. 2009). Hepatocytes are sensitive to the oxidative stress induced by CCl_4_ metabolites and cytokines that cause an inflammatory response.

Additionally, acute or chronic CCl_4_-induced hepatotoxicity causes **a)** activation on cytochrome P-450 2E1 (CYP2E1), **b)** generation of reactive metabolites such as trichloromethyl radicals (CCl_3_), and **c)** peroxy trichloromethyl radicals (OOCCl_3_). These radicals bind covalently to macromolecules such as lipids, proteins, and nucleic acids (Khan et al. 2012). Polyunsaturated fatty acids are sensitive to oxidation by free radicals. Thus, CCl_4_ metabolites promote lipid peroxidation that increases the levels of lipoperoxide and free peroxide radicals, resulting in liver injury or necrosis.

*Tagetes lucida* Cav. (Asteraceae) is native to Central America. Its common names include sweet-scented marigold, Mexican mint marigold, Mexican tarragon, sweet mace, and Texas tarragon. *T. lucida* is new in Egypt. *T. lucida* plant reaches 18-30 inches in height, with a branched stem and linear to oblong leaves of shiny medium green. The plant’s small golden-yellow flowers appear in late summer at the ends of the stems. Traditionally, *T. lucida* has been prescribed to treat fever, tumours, diarrhoea, asthma, rheumatism, and flu. Also, it has been recommended to treat nervous complaints, including anxiety, irritability, and depression (Gabriela et al. 2012). Various studies demonstrated the biological activities of *T. lucida* as platelet anti-aggregate, nematicide and antimicrobial (Céspedes et al. 2006), anti-inflammatory (Sepúlveda-Arias et al. 2013), antidepressant (Bonilla-Jaime et al. 2015), hepatoprotective (El-Newary et al. 2016), and antihyperglycemic (Abdel-Haleem et al. 2017). Several activities of *T. lucida* roots have been documented, including antimicrobial and antifungal (Damian-Badillo et al. 2008). Vega-Avila et al. (2009**)** demonstrated that the roots of *T. lucida* are cytotoxic to crustaceans, with low LC_50_ values due to the high content of coumarins in the roots. Many valuable components occur in *T. lucida* plants as essential oils, polyphenols, flavonoids, and coumarins (Céspedes et al. 2006).

This work aimed to study the effect of *T. lucida* roots ethanol extract (TLRE) on CCl_4_-induced hepatotoxicity in rats.

## Materials and methods

### Collection of plant material and preparation of extract

Seeds of *T. lucida* were imported from the Canadian branch of Johnny's Selected Seeds www.johnnyseeds.com (product ID: 2273) in 2017. Seeds were cultivated at the SEKM Company Farm at Bilbase, El-Sharkya Governorate, Egypt, during the winter (January) of 2018. Plants were collected in May at the flowering stage, and the roots were separated. The cultivated plant was verified by the National Research Centre herbarium (CAIRC) team. The plant was deposited in the herbarium under Voucher Specimen No. M132. Roots were exposed to heat shock at 100 °C for 1 min to stop enzyme activity that might convert phytochemicals, and they were then air-dried in the shade to completion in an oven at 40 °C. Powdered roots (1000 g) were exhaustively extracted with 70% ethanol (EtOH) by soaking at room temperature. The extract was lyophilised, and the resulting powder was kept at −20 °C until use (El-Newary et al. 2016).

### Chemical composition of TLRE

#### Quantitative analysis

The total phenolic content of TLRE was determined by the Folin-Ciocalteu method (Gorinstein et al. 2004). The total phenolic content was expressed as gallic acid equivalents (mg gallic acid/g dry weight). The total flavonoid content of TLRE was determined according to the method of Lin and Tang (2007). The total flavonoid content was expressed in terms of quercetin. TLRE crude alkaloids were gravimetrically determined according to the procedure proposed by Onwuka (2006). The total coumarin content of TLRE was determined according to the method of Nassar (1986**)** and was expressed as umbelliferone.

#### HPLC analysis of phenolic compounds

Phenols of TLRE were identified by high-performance liquid chromatography (HPLC) analysis, according to Kim et al. (2006), using an Agilent Technologies 1260 series device. The separation was carried out using a C18 column (4.6 mm × 250 mm i.d., 5 μm). The mobile phase consisted of water (A) and acetonitrile (B) at a flow rate of 1 mL/min. The mobile phase was programmed consecutively in a linear gradient as follows: 0 min (80% A); 0–5 min (80% A); 5–8 min (40% A); 8–12 min (50% A); 12–14 min (80% A); and 14–16 min (80% A). A multiple-wavelength detector monitored the process at 280 nm. An injection volume of 10 μL was used for each sample solution. The column temperature was maintained at 35 °C.

#### Paper chromatography analysis of TLRE

The HPLC analysis did not identify the major component in TLRE, so TLRE was evaluated by 2-dimensional paper chromatography. The diluted TLRE (1 mL) was spotted in the corner of a 20 × 20 cm paper chromatography sheet (Whatman No. 1) about 2 cm from the edges. The separation was first developed with a nonpolar running system after air-drying (chloroform: methanol, 3:1), followed by a polar running system (acetonitrile: methanol: 3:1) the direction of the nonpolar run. The paper sheet was allowed to dry at room temperature and then was visualised with ultraviolet (UV) light at 354 nm; the major compound appeared as a pale bright yellow spot at the corner. After detecting the spot, it was cut out and re-extracted from the paper with 99% ethanol; after concentration, the residue was subjected to structure analysis by infra-red (IR) and mass (MS) spectrometry.

### Determination of acute toxicity (LD_50_)

The toxicity of TLRE was evaluated in Swiss albino mice (*n* = 8) by the method of Bruce (1985). Rats received serial doses orally of TLRE to be tested, at 500, 1000, 2000, 3000, 4000, and 5000 mg/kg body weight, while the control group received only normal saline. The mortality in all groups was observed for 48 h for assessing toxicity. Living animals were observed for 2 weeks. The mortality number for each concentration during the first 48 h was entered into the BioStat program (BioStat 2009 Build 5.8.4.3), and the LD_50_ of the extract was calculated as 5000 mg/kg body weight.

### Studying the therapeutic and protective effect of TLRE versus CCl_4_-induced hepatotoxicity in rats

#### Animals and animal care

This study was approved by the Medical Research Ethics Committee, National Research Centre, Egypt, under registration no. 19/095.

Male albino Wistar strain rats (90 rats) weighing 140–180 g each were obtained from the central animal house of the National Research Centre, Dokki, Giza, Egypt. Animals were housed in plastic cages under laboratory conditions (25 ± 5 °C, 65 ± 5% humidity, 10–12 h light/dark cycle) at the animal facility of the animal house. The feed was a commercial chow-based diet obtained from the animal house. Water and food were available *ad libitum* for the ten days of study.

#### Hepatotoxicity induction

CCl_4_-induced hepatotoxicity model was used in this study according to Mir et al. (2010) with slight modification. Rats administrated CCl_4_ at 2.00 mL/kg body weight orally as a single dose. The CCl_4_ liquid solution was dissolved in corn oil (1:1), where each mL of CCl_4_ mixed with 1 mL of corn oil.

#### Dosing and standard protocol

Based on the work of Garg et al. (2016) and Ghosh (1984**),** the TLRE dose was calculated as 1/10 of the LD_50_ that was 500 mg/kg/day. The dose of silymarin was the standard recommended dose, 25 mg/kg/day (Ibrahim et al. 2010). For treatment, TLRE or silymarin was dissolved in normal saline 0.9%. Rats were force-fed TLRE or silymarin orally for 7 days.

#### Experimental design

The rats were randomly divided into four main groups.

The first main group was the negative group (n = 10), which received a saline solution for the entire experimental period. This group was maintained as a negative control.

The second main group was the positive group (20 rats), broken into two subgroups; each subgroup of 10 rats received either silymarin or TLRE, respectively. These were maintained as the positive control for the two materials. Rats in these subgroups were killed on the 10^th^ day of the experiment.

The third main group was the therapeutic group (30 rats), which was designed to evaluate the therapeutic effect of TLRE, according to Ibrahim et al. (2014). The rats in this main group were firstly force-fed CCl_4_ at 2 mL/kg body weight as a single dose, and after two days, these rats were treated with either the TLRE or silymarin for 7 days. These rats were killed on the 10^th^ day. The therapeutic group was classified into three subgroups (n = 10). Rats received saline only for 7 days and were maintained as the therapeutic control. Rats received the recommended dose of silymarin for 7 days, and rats received the TLRE 500 mg/kg body weight for 7 days.

The fourth main group was the hepatoprotective group (30 rats) that was designed to evaluate the hepatoprotective effect of TLRE, according to Ibrahim et al. (2014). Rats in the hepatoprotective group firstly received the silymarin or TLRE or saline solution for 7 days, and then these rats were force-fed with CCl_4_ at 2 mL/kg body weight as a single dose. These rats were killed two days later, on the tenth day. This hepatoprotective group was divided into three subgroups (n = 10). First, rats received saline only for 7 days and were maintained as a hepatoprotective control. Rats received the recommended dose of silymarin for 7 days, and rats received the extract as 500 mg/kg body weight for 7 days.

At the end of the experimental period, rats were fasted overnight and injected with 87 mg ketamine/kg body weight and 13 mg xylazine/kg body weight for anaesthesia. The two drugs were dissolved in normal saline, and each rat received 0.2 mL/100 g body weight of the combined solution (Van Pelt 1977). Under anaesthesia, animals were sacrificed, and blood samples were collected from the retro-orbital plexus. Blood was centrifuged (4000 *g*, 10 min, 4 °C, using a Sigma Laboratory centrifuge) to separate the serum. Livers were separated, washed in ice-cold 1.15% KCl solution, and freshly weighed. A piece of liver from each rat was homogenised in ice-cold Tris-HCl buffer, 0.1 M, pH 7.4, using an ultrasonic homogeniser. The homogenate was centrifuged at 4000 *g* for 15 min at 4 °C using a Sigma Laboratory centrifuge and kept at −20 °C until analysis of the antioxidants. Another piece of liver from each rat was removed, and the liver microsomes were prepared and were isolated according to a previous method (Lowry et al. 1951). A piece of liver from each rat was also stored in 10% formalin for histopathological testing.

#### Estimation of biochemical parameters

The liver functions of the rats were determined spectrophotometrically in serum using commercial kits. Total protein (TP), albumin, total bilirubin, and aspartate aminotransferase (AST) and alanine aminotransferase (ALT), and alkaline phosphatase (ALP) activities were measured according to Henry (1964), Dumas et al. (1997), Reitman and Frankel (1957), and Belfield and Goldberg (1969**)**. In addition, globulin was calculated as the difference between TP and albumin according to the method of Reinhold (1953).

Liver antioxidant enzyme activities were determined in the liver homogenate. Activities of catalase (CAT), superoxide dismutase (SOD), glutathione reductase (GR), and glutathione peroxidase (GPx) were determined according to the methods described by Paglia and Valentine (1967), Beers and Sizer (1952), Fridovich (1974), Habig et al. (1974) and Goldberg and Spooner (1983). Also, glutathione (GSH), as a nonenzymatic antioxidant, was determined spectrophotometrically according to Griffith (1980).

Oxidative stress biomarkers were determined in the serum and liver homogenate samples. Malondialdehyde (MDA) is a lipid peroxidation biomarker and was assayed by a method described by Ohkawa et al. (1979). The concentration of hydrogen peroxide (H_2_O_2_) in liver homogenate was estimated according to Chance and Maehly (1955).

CYP2E1 activity was measured in the liver microsomal fraction as described by Chang et al. (2006**)**. TNF-α content was determined using an enzyme-linked immunosorbent assay kit; the instructions from the manufacturer, NOVA kit, Beijing, China, were followed to calculate the results.

Liver total lipid (TL), total cholesterol (TC), and triglycerides (TG) were extracted using the procedure developed by Folch et al. (1957). The concentrations of TC and TG in the liver were analysed with the same enzymatic kits as used in the serum analysis.

Some of the data were expressed as an inhibition percentage; IR% = [(control value - treated value)/control value] × 100.

#### Histopathological studies

Specimens of all animals were dissected immediately after death and fixed in 10% neutral-buffered formal saline for at least 72 h. All the samples were washed in tap water for half an hour and then dehydrated in ascending grades of alcohol (70%, 80%, 90%, and finally absolute alcohol), cleared in xylene, impregnated in soft paraffin at 55 °C, and embedded in hard paraffin. Serial sections were cut at 6 µm thickness and stained with haematoxylin and eosin (Drury and Wallington 1980) for histopathological investigation.

### Statistical analysis

Data were analysed as mean ± SE for eight rats each. Comparisons among groups were performed by one-way analysis of variance ANOVA test at *P* ≤ 0.05 followed by Tukey comparison test using IBM-SPSS (version 25) followed by a *post hoc* test.

## Results

### Chemical composition of TLRE

**Figure 1.**
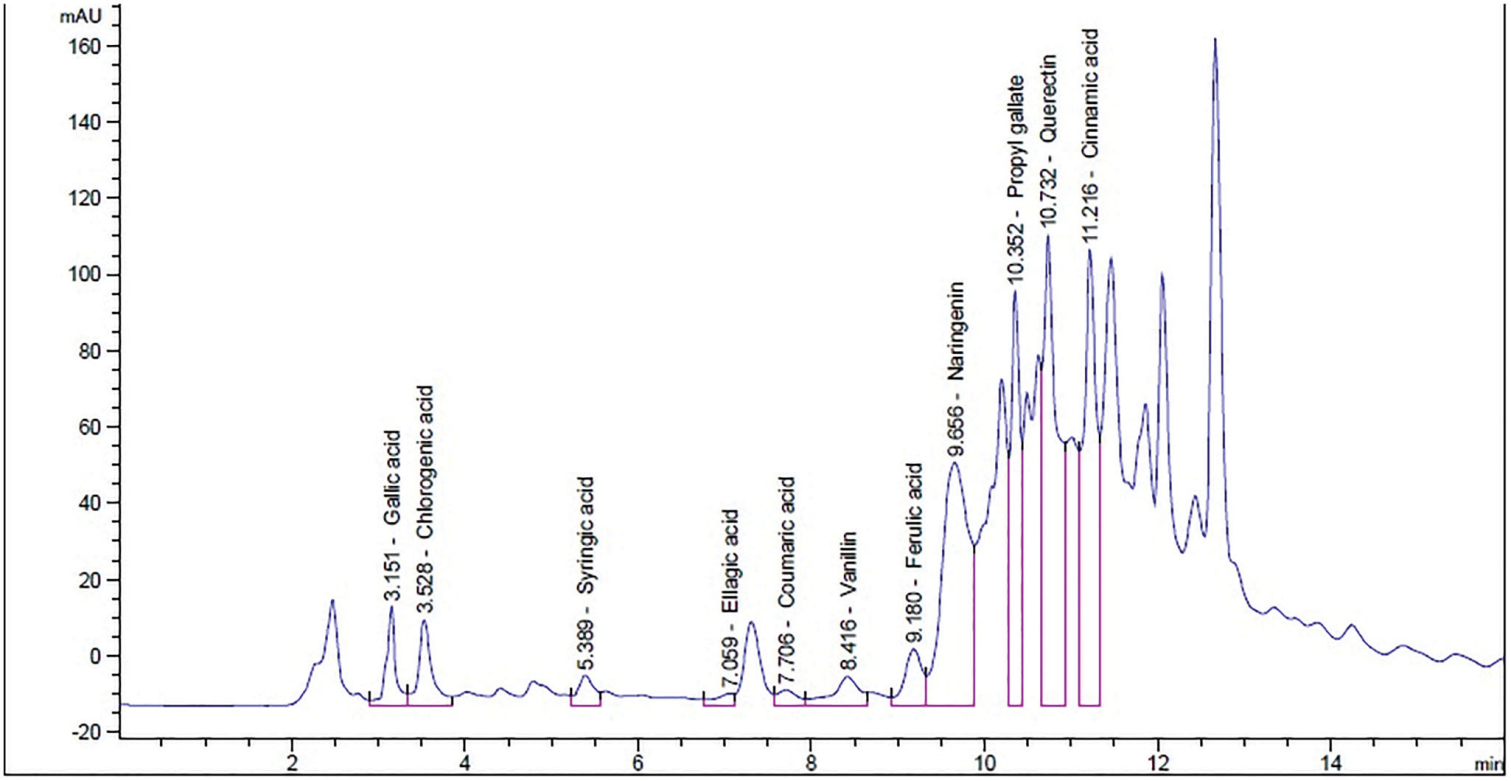
The chemical composition of *Tagetes lucida* roots extract as analysed by the HPLC. *mAU means a milli-absorbance unit or 0.001 absorbance units that are used to measure absorbance.

In this study, TLRE contains 12.62 ± 1.21 mg polyphenols as gallic acid/g extract, 5.64 ± 0.44 mg flavonoids as quercetin/g, 9.55 ± 0.15 mg coumarins as umbelliferone/g extract, and 238.00 ± 1.77 mg total alkaloids/g extract. The phytochemical composition of TLRE was identified from the HPLC analysis. The identified compounds include gallic acid (399.76 µg/g extract), chlorogenic acid (1426.04 µg/g extract), syringic acid (143.80 µg/g extract), ellagic acid (180.25 µg/g extract), coumaric acid (58.74 µg/g extract), ferulic acid (208.22 µg/g extract), cinnamic acid (408.94 µg/g extract), propyl gallate (670.08 µg/g extract), vanillin (198.18 µg/g extract), naringenin (1419.65 µg/g extract), and quercetin (4182.24 µg/g extract) (Figure 1).

**Figure 2.**
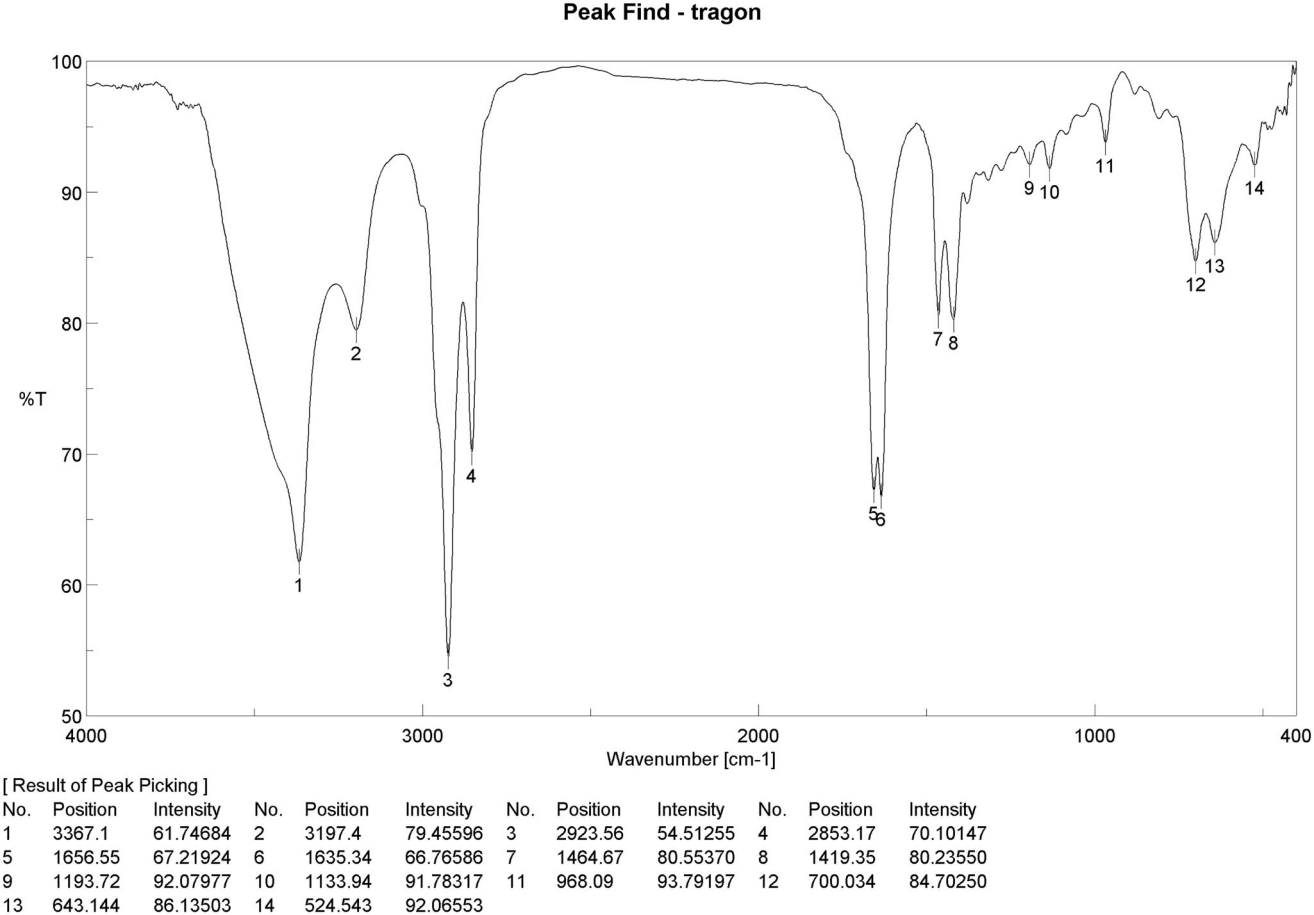
Identification of corynan-17-ol, 18,19-didehydro-10-methoxy-, acetate (ester), the major component of *Tagetes lucida* root extract; IR. *Y axis; T% means transition percentage, X axis; was wavenumber band (cm^−1^).

Infra-red (IR) absorption bands at 3367, 3167, 1656, 1635, and 1464 cm^−1^ suggested the presence of NH, OH, aromatic ring, and ester carbonyl functionalities. The C-N stretching and C-N bending vibrations of a nitro group occur near 968 cm^−1^ and 700 cm^−1^, respectively (Gopathy et al. 2019) (Figure 2).

**Figure 3.**
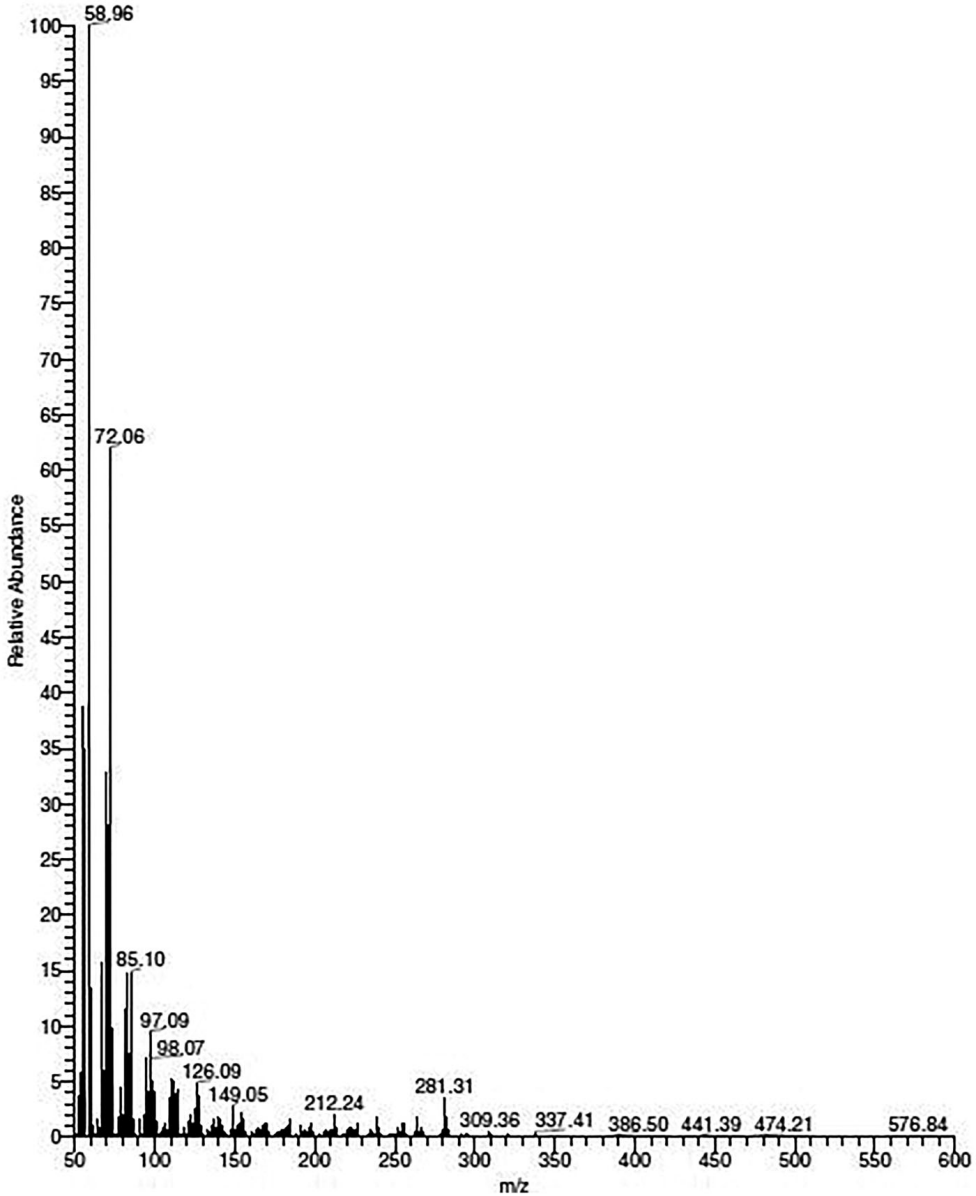
Identification of corynan-17-ol, 18,19-didehydro-10-methoxy-, acetate (ester), the major component of *Tagetes lucida* root extract; MS. *m/z *means mass/charge.

**Figure 4.**
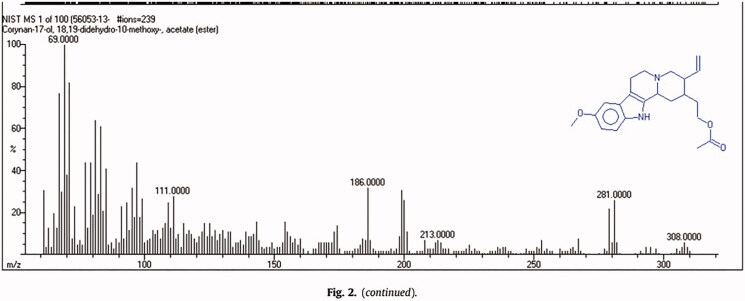
Identification of corynan-17-ol, 18,19-didehydro-10-methoxy-, acetate (ester), the major component of *Tagetes lucida* root extract. Corynan-17-ol, 18,19-didehydro-10-methoxy-, acetate (ester) 17-(acetyloxy)-18,19-didehydro-10-methoxycorynan CAS NO: 56053-13-5. Molecular Formula: C22H28N2O3 and Molecular Weight: 368.46932. *m/z *means mass/charge.

Mass spectroscopy produced peaks at *m/z* 308 and 281, referred to as the indole-alkaloid skeleton (Zhang et al. 2017) (Figure 3). The mass spectrum of the compound was compared in terms of *m/z* peak similarity using the European MassBank (NORMAN MassBank) database, https://massbank.eu/MassBank, and the MassBank of North America database, https://mona.fiehnlab.ucdavis.edu. Structure confirmation was carried out using NIST mass search software and suggested that the structure was corynan-17-ol, 18,19-didehydro-10-methyoxy, with a molecular formula C_21_H_24_N_2_O_4_ (Figure 4) (Bhagavathy et al. 2019).

### The protective and therapeutic effect of TLRE against CCl_4_-induced hepatotoxicity in rats

#### Influence of TLRE on total body weight gain

The data presented in Table 1 show that the therapeutic rats that were firstly force-fed CCl_4_ increased in body weight by only 10.22 ± 2.23 g during the experimental period. Also, the protective group of rats, which received CCl_4_ as the final treatment, did not add weight but lost about 7.03 ± 1.12 g from their body weight, compared to the total body weight gain of the negative control (33.90 ± 3.69 g), *p* ≤ 0.05.

**Table 1.** The therapeutic and protective effect of *Tagetes lucida* root extract on total body weight gain (g) and relative weight of liver (%), CYP2E1 activity, and TNF-α content.

Parameters Groups		Total body weight gain	Relative weight of liver	CYP2E1 activity (nm/min/mg protein)	TNF-α content (pg/ mg protein)
Negative group	(ve- control)	33.90 ± 3.69^c^	4.42 ± 0.18^c^	3.08 ± 0.29^f^	15.2 ± 0.55^d^
Positive groups	ve + Silymarin	40.48 ± 3.18^b^	3.92 ± 0.30^de^	2.80 ± 0.31^f^	14.1 ± 0.99^d^
	*ve + T. lucida*	45.73 ± 2.77^a^	3.62 ± 0.20^e^	2.58 ± 0.56^f^	14.44 ± 0.89^d^
Therapeutic group	CCl_4_ Control	10.22 ± 2.23^de^	4.34 ± 0.20^cd^	11.27 ± 0.78^a^	38.83 ± 2.30^a^
	Silymarin	10.86 ± 1.81^de^	3.71 ± 0.28^e^	6.43 ± 0.35^d^	17.56 ± 1.34^c^
	T. lucida	11.54 ± 0.78^de^	3.94 ± 0.35^de^	6.87 ± 0.49^c^	17.97 ± 0.57^c^
protective group	CCl_4_ Control	-7.03 ± 1.21^f^	5.25 ± 0.30^a^	9.08 ± 0.56^b^	30.01 ± 2.34^b^
	Silymarin	8.02 ± 2.87^e^	4.88 ± 0.25^b^	4.55 ± 0.27^e^	13.97 ± 1.32^d^
	T. lucida	14.88 ± 1.28^d^	4.78 ± 0.30^b^	4.70 ± 0.53^e^	13.89 ± 1.34^d^

Data are presented as the means ± S.D of eight replicates. Data analysed by ANOVA one-way, *p* ≤ 0.05, Value with the same letter has no significant but value with different letter has significant at 0.05.

TLRE increased the total body weight gain of the therapeutic rats, but silymarin did not significantly change it compared to the therapeutic control group (*p* ≤ 0.05). In the protective control rats, TLRE prevented the weight loss and increased the total body weight gain of these rats to 14.88 ± 1.28 g, compared to silymarin treatment, which increased the body weight gain 8.02 ± 0.87 g during the experimental period (*p* ≤ 0.05). In addition, the total body weight gain for the positive control of TLRE or silymarin was elevated compared to that recorded in the negative control group. Thus, the effect of TLRE on total body weight gain in the therapeutic and protective rats was close to that of silymarin treatment.

#### Influence of TLRE on the relative weight of the liver

The relative weight of the liver of protective rats was raised by 5.25 ± 0.3%, significant compared with that of the negative control, 4.42 ± 0.18% (*p* ≤ 0.05) **(**Table 1**)**. The relative weight of the liver of therapeutic rats force-fed with either TLRE or silymarin was significantly reduced to 3.71 ± 0.28 and 3.94 ± 0.35%, respectively, compared to the value in the therapeutic control 4.34 ± 0.2% (*p* ≤ 0. 05). Either TLRE or silymarin protected the liver of protective-group rats, where the relative weight of the liver declined to 4.78 ± 0.3% and 4.88 ± 0.25%, respectively, compared to the value for the protective control 5.25 ± 0.3% (*p* ≤ 0.05). Compared to the negative control, the relative weight of the liver of the positive control for TLRE and silymarin was significantly reduced.

#### Influence of TLRE on CYP2E1 activity and TNF-α content

CYT2E1 level was elevated to 11.27 ± 0.78 and 9.08 ± 0.56 nmol/min/mg protein in the therapeutic and protective control group, respectively, which was significant in comparison with the level in the negative control, 3.08 ± 0.29 nmol/min/mg protein (*p* ≤ 0.05) (Table 1). TLRE recorded an ameliorative effect on CYT2E1 expression, showing a significant decrease in the therapeutic (6.87 ± 0.49 nmol/min/mg protein) and the protective (4.70 ± 0.53 nmol/min/mg protein) groups, compared to the corresponding control. The CYT2E1 level in the positive group for TLRE did not significantly change from that of the negative control. No significant differences were noticed between the levels of CYT2E1 in rats treated with TLRE or silymarin in each main group.

The CCl_4_ administration caused remarkable inflammation, where TNF-α content significantly increased from 15.20 ± 0.55 pg/mg protein in the negative control rats to 38.83 ± 2.80 pg/mg protein in the therapeutic control and 30.01 ± 2.34 pg/mg protein in the protective control (*p* ≤ 0.05) (Table. 1). Treatment with TLRE restored inflammation status towards the normal rate, as evidenced by a reduction in TNF-α content to 17.97 ± 0.57 pg/mg protein and 13.89 ± 1.34 pg/mg protein therapeutic protective group, respectively, which was significant compared to each control. The TNF-α content for the positive controls remained close to that in the negative control group. The ameliorative effect of TLRE on TNF-α was close to that recorded for silymarin.

#### Influence of TLRE on liver functions

This study examined liver performance evaluated by liver function, which was dramatically affected by CCl_4_ administration (Table 2). Hepatocytes of the rats force-fed CCl_4_ in either the therapeutic or the protective controls released AST and ALT enzymes in the serum at higher levels than those released in the negative control group. Also, these rats produced ALP enzyme and bilirubin in serum at higher levels than those produced in the negative control. The TP level of rats in the therapeutic control that first received CCl_4_ did not change significantly. In contrast, the TP for the protective control group was significantly reduced compared to that of the negative control (*p* ≤ 0.05). In addition, albumin in CCl_4_-treated rats declined considerably in comparison with the level in the negative control.

**Table 2.** Effect of *Tagetes lucida* root extract on liver functions of CCl_4_-induced hepatotoxicity in rats.

Main groups	Subgroups	TP mg/dl	Alb mg/dl	Glo mg/dl	Alb/Glo %	T. bilirubin mg/dl	AST U/L	ALT U/L	ALP mg/dl
Negative group Positive group	ve^-^ control	7.60^c^ ± 0.32	3.47^bc^ ± 0.16	4.13^cd^ ± 0.25	0.85^a^ ± 0.10	0.99^c^ ± 0.08	75.70^d^ ± 3.42	66.64^d^ ± 3.27	143.12^g^ ± 2.64
	ve^+^ Silymarin	7.58^c^ ± 0.32	3.45^bc^ ± 0.09	4.13^cd^ ± 0.20	0.84^a^ ± 0.04	0.99^c^ ± 0.07	77.78^d^ ± 2.84	68.65^d^ ± 4.00	141.38^g^ ± 4.46
	ve^+^ *T. lucida*	8.47^ab^ ± 0.27	4.04^abb^ ± 0.33	4.43^bc^ ± 0.18	0.92^a^ ± 0.11	0.81^c^ ± 0.03	76.97^d^ ± 1.91	52.30^e^ ± 1.97	136.26^h^ ± 2.48
Therapeutic group	CCl_4_ Control	7.66^c^ ± 0.19	3.29^c^ ± 0.12	4.37^bc^ ± 0.20	0.75^a^ ± 0.05	1.19^b^ ± 0.09	85.96^c^ ± 1.50	77.47^c^ ± 1.54	196.10^c^ ± 5.68
	Silymarin	8.62^a^ ± 0.29	3.83^ab^ ± 0.26	4.80^a^ ± 0.23	0.80^a^ ± 0.09	0.92^c^ ± 0.08	79.19^d^ ± 3.35	50.12^e^ ± 2.80	181.15^e^ ± 2.47
	*T. lucida*	7.80^bc^ ± 0.24	3.51^bc^ ± 0.31	4.29^bc^ ± 0.24	0.82^a^ ± 0.11	0.85^c^ ± 0.07	75.24^d^ ± 3.01	53.86^e^ ± 3.22	162.22^f^ ± 4.33
Protective group	CCl_4_ Control	6.70^d^ ± 0.18	2.91^d^ ± 0.33	3.79^d^ ± 0.25	0.77^a^ ± 0.13	2.06^a^ ± 0.18	122.21^a^ ± 2.25	238.61^a^ ± 4.62	226.21^a^ ± 3.62
	Silymarin	8.00^abc^ ± 0.73	3.61^bc^ ± 0.04	4.39^bc^ ± 0.26	0.83^a^ ± 0.03	1.20^b^ ± 0.09	112.51^b^ ± 3.45	213.74^b^ ± 4.72	207.31^b^ ± 2.87
	*T. lucida*	8.30^abc^ ± 0.58	3.62^bc^ ± 0.29	4.68^ab^ ± 0.34	0.78^a^ ± 0.08	1.18^b^ ± 0.11	114.80^b^ ± 3.54	211.44^b^ ± 5.65	190.25^d^ ± 3.52

Data are presented as the means  ±  S.D of eight replicates. Data analysed by ANOVA one-way, *p* ≤ 0.05, Value with the same letter has no significant but value with different letter has significant at 0.05.

TLRE as a therapeutic agent did not cause a significant effect on TP, albumin, and globulin concentrations compared to the corresponding values in the therapeutic control. Meanwhile, TLRE as a protective agent protected hepatocytes and maintained TP and its fractions at the normal levels (Table 2). Thus, TP, albumin, and globulin concentrations were significantly elevated, from 6.70 ± 0.18, 2.91 ± 0.33, and 3.79 ± 0.25 g/dL, respectively, in the protective control to 8.30 ± 0.58, 3.62 ± 0.29, and 4.68 ± 0.34 g/dL, respectively (*p* ≤ 0.05). TLRE recovered and protected TP and its fractions at levels close to the effects of silymarin.

TLRE decreased the total bilirubin level of therapeutic rats, to 0.85 ± 0.07 mg/dL, compared to therapeutic control (1.19 ± 0.09 mg/dL). Also, the ALP activity of the therapeutic rats was reduced to 162.22 ± 4.33 U/L, a significant change compared to the therapeutic control. In the protective group, TLRE maintained bilirubin concentration and ALP activity lower than the protective control (*p* ≤ 0.05). The effect of TLRE on total bilirubin concentration and ALP activity was close to the effect of silymarin. The bilirubin level of the positive control for TLRE did not change significantly; however, the ALP activity was significantly reduced compared to that of the negative control.

The activity of AST and ALT enzymes in serum was significantly lowered due to the administration of TLRE as a therapeutic agent (Table 2). The AST and ALT levels were significantly reduced, from 85.96 ± 1.50 and 77.47 ± 1.54 U/L, respectively, in the therapeutic control to 75.24 ± 3.01 and 53.86 ± 3.22 U/L as a response to TLRE administration (*p ≤* 0.05). Also, TLRE exhibited a protective effect on AST and ALT close to the effect produced by silymarin. The AST activity of TLRE positive control did not change significantly compared to that of the negative control; however, the ALT level was reduced considerably to a level lower than for the ALT of the negative control.

#### Influence of TLRE on oxidative stress biomarkers

Treatment with CCl_4_ caused remarkable oxidative stress status that appeared as an increment in H_2_O_2_ production in the therapeutic and protective controls: 6.65 ± 0.25 and 6.49 ± 0.29 μmol/g, significant when compared to the concentration in the negative control, 3.55 ± 0.27 μmol/g (*p* ≤ 0. 05) (Table 3). In addition, the MDA levels in liver tissue and serum of the therapeutic and protective controls were significantly elevated compared to the MDA levels for the negative control (*p* ≤ 0.05).

**Table 3.** Effect of *Tagetes lucida* root extract on oxidative stress biomarkers and antioxidant biomarkers.

		Oxidative stress biomarkers			Antioxidant biomarkers					
Parameters Groups		*H_2_O_2_ (uM/ g tissue)*	*MDA (nmol/ g tissue)*	*MDA (nmol/ ml serum)*	CAT (U/ mg protein)	SOD U/mg protein	GSH mg/dL	GR U/mg protein	GST U/mg protein	GPx U/mg protein
*Negative group (^-^ve Control )*		3.55 ± 0.27^b^	14.93 ± 0.69^d^	7.35 ± 0.15^d^	5.78 ± 0.47^e^	9.50 ± 0.23^d^	13.51 ± 1.30^ef^	12.37 ± 2.12^c^	10.25 ± 1.58^c^	7.33 ± 0.47^d^
*Positive group*	*+ve Silymarin*	3.86 ± 0.36^b^	12.04 ± 1.32^e^	7.26 ± 0.13^d^	5.79 ± 0.32^e^	9.67 ± 0.57^d^	14.38 ± 0.69^de^	13.44 ± 1.00^c^	11.25 ± 1.65^c^	7.38 ± 0.30^d^
	*+ve T. lucida extract*	3.52 ± 0.31^b^	10.64 ± 0.83^f^	7.67 ± 0.16 ^cd^	6.12 ± 0.39^e^	12.96 ± 0.96^a^	14.72 ± 1.82^d^	13.03 ± 1.51^c^	11.04 ± 1.27^c^	9.51 ± 0.93^d^
*Therapeutic group*	*CCl_4_ Control*	6.65 ± 0.25^a^	24.46 ± 1.63^b^	10.81 ± 0.48^a^	4.45 ± 0.23^f^	6.64 ± 0.43^f^	5.58 ± 0.64^g^	4.86 ± 0.03^d^	4.17 ± 0.26^d^	3.72 ± 0.33^e^
	*Therapeutic Silymarin*	3.89 ± 0.18^b^	14.76 ± 1.16^d^	7.97 ± 0.29^c^	8.46 ± 0.64^b^	10.55 ± 0.47^c^	18.58 ± 1.23^a^	17.82 ± 1.01^a^	15.25 ± 0.87^a^	10.38 ± 1.39^a^
	*Therapeutic extract*	3.57 ± 0.27^b^	18.06 ± 0.85^c^	8.03 ± 0.48^c^	10.00 ± 0.56^a^	11.45 ± 0.81^b^	16.61 ± 0.99^c^	15.83 ± 1.53^b^	14.17 ± 1.37^b^	10.30 ± 0.43^a^
*protective group*	*CCl_4_ protective Control*	6.49 ± 0.29^a^	33.63 ± 1.68^a^	9.22 ± 0.27^b^	4.69 ± 0.13^f^	7.43 ± 0.59^e^	3.37 ± 0.77^h^	2.69 ± 0.52^e^	2.36 ± 0.45^e^	2.18 ± 0.18^f^
	*protective Silymarin*	3.07 ± 0.26^c^	15.37 ± 0.85^d^	7.6 ± 0.211^cd^	6.44 ± 0.34^d^	10.56 ± 0.86^c^	13.22 ± 1.46^f^	12.94 ± 1.17^c^	11.37 ± 1.02^c^	9.14 ± 0.29^bc^
	*protective extract*	3.06 ± 0.21^c^	17.03 ± 0.74^c^	7.65 ± 0.33 ^cd^	7.47 ± 0.54^c^	11.62 ± 0.39^b^	17.62 ± 1.74^b^	16.99 ± 1.14^a^	14.70 ± 0.99^ab^	8.67 ± 0.37^c^

Data are presented as the means ± S.D of eight replicates. Data analysed by ANOVA one-way, P ≤ 0.05, Value with the same letter has no significant but value with different letter has significant at 0.05. H_2_O_2_: hydrogen peroxide; MDA: malondialdehyde the lipid peroxidation biomarker; CAT: catalase; SOD: superoxide dismutase; GSH: glutathione reduced; GR: glutathione reductase; GST: glutathione S transferase; GPx: glutathione peroxidase.

The TLRE, either as a therapeutic agent or as a protective agent, ameliorated the oxidative stress status. The H_2_O_2_ production in the liver tissue was significantly reduced to 3.57 ± 0.27 and 3.06 ± 0.21 μmol/g tissue, respectively, compared to the corresponding control. H_2_O_2_ production in positive control rats did not change significantly compared to the negative control. In comparison with silymarin, TLRE exhibited potent therapeutic and protective activities. No significant difference was recorded in H_2_O_2_ production between the rats treated with TLRE or silymarin and the negative control.

The levels of MDA of the therapeutic rats, whether in the liver tissues or released into the serum, were significantly reduced to 18.06 ± 0.85 nmol/g and 8.03 ± 0.48 nmol/mL, respectively, compared to the MDA levels for the therapeutic control of 24.46 ± 1.63 nmol/g and 10.81 ± 0.48 nmol/mL (*p* ≤ 0. 05). In the protective group, the same trend took place. TLRE caused a decrease in MDA concentration in the liver and serum, reaching 17.03 ± 0.74 nmol/g and 7.65 ± 0.33 nmol/mL, respectively, which was significant compared to the protective control (33.63 ± 1.68 nmol/g and 9.22 ± 0.27 nmol/mL, respectively). No significant difference was noticed between the levels of MDA of the serum or the liver of the positive control rats and those found in the negative control rats.

#### Influence of TLRE on antioxidant biomarkers in the liver

Oxidative stress conditions that were recorded as a response to the CCl_4_ force-feeding represented as a significant reduction in enzymatic antioxidants, including the activities of GR, GST, GPx, CAT, and SOD, as well as for a nonenzymatic antioxidant, the GSH content, in comparison with the values for the negative control (Table 3).

Administration TLRE attenuated CCl_4_-induced liver injury in the therapeutic and protective group exhibited a significant increase in CAT activity to 10.00 ± 0.56 and 7.47 ± 0.54 U/mg protein, respectively, compared to the corresponding control (*p* ≤ 0.05). The recovery effect on CAT activity produced by TLRE was better than that from silymarin in the therapeutic and protective groups. The CAT activity of the positive control did not alter significantly compared to the CAT activity of the negative control. TLRE improved the SOD activity in the therapeutic and protective groups, where the activity increased dramatically by about 72.44 and 56.39% compared to the values for the corresponding control. The effect of TLRE on SOD activity was close to the result shown with silymarin. TLRE did not affect the SOD activity of the positive control rats.

The administration of TLRE improved CCl_4_-induced injury to the liver glutathione system for the therapeutic and protective groups compared to effects in each control (*p* ≤ 0.05). The GSH content and related enzymes GR, GST, and GPx of the therapeutic group were significantly elevated to 16.61 ± 0.99 mg/dL, 15.83 ± 1.53, 14.17 ± 1.37, and 10.30 ± 0.43 U/mg protein, respectively, as a response to TLRE treatment, compared to levels in the therapeutic control. Meanwhile, TLRE prevented imbalance in the GSH, GR, GST, and GPx levels in the protective group of rats and maintained them close to the normal levels at 17.62 ± 1.74 mg/dL, 16.99 ± 1.14, 14.70 ± 0.99, and 8.67 ± 0.37 U/mg protein, respectively, compared to the protective control group. TLRE did not alter the glutathione system of the rats in the positive control group compared to the levels of the negative control group.

#### The effects on the lipid profile of the liver

The CCl_4_-induced hepatotoxicity taking place in the rat liver increased the lipid accumulation in the liver, shown as total lipid (TL), total cholesterol (TC), and triglycerides (TG) of the therapeutic and protective control, compared to the levels for the negative control (*p* ≤ 0. 05), as shown in Table 4. TLRE showed a hypolipidemic effect demonstrated by a significant reduction in TL, TC, and TG. TLRE significantly reduced the TL, TC, and TG in the therapeutic group by about 10.68, 49.85, and 54.22%, consequently, compared to the therapeutic control (*p* ≤ 0.05). TLRE exhibited an anti-hyperlipidemia effect in the protective group of rats and prevented lipid accumulation in the liver there. TLRE restored the TL, TC, and TG of the protective group to within normal levels. Thus, TL, TC, and TG parameters were significantly decreased by about 33.98, 41.90, and 34.71%, respectively, compared with the corresponding values in the protective control (*p* ≤ 0.05). The positive group appeared with a normal liver lipid profile, where the TL, TC, and TG levels were decreased or did not change compared to the same parameters in the negative control. The improvements in liver lipid profile recorded in the silymarin-treated groups were close to those recorded in TLRE-treated groups.

**Table 4.** The effect of *Tagetes lucida* roots alcoholic extract in lipid profile of liver.

Groups Parameters		T lipid (mg/g liver)	TC (mg/g liver)	TG (mg/g liver)
Negative group (ve- control)		90.10 ± 8.47^d^	30.86 ± 0.55^c^	49.05 ± 2.06^c^
Positive group	ve + Silymarin	83.44 ± 3.58^e^	28.36 ± 2.93^d^	47.60 ± 2.24^c^
	ve*+ T. lucida*	89.97 ± 5.52^d^	26.11 ± 2.60^e^	46.80 ± 1.57^c^
Therapeutic group	*CCl_4_ Control*	105.76 ± 5.72^b^	36.97 ± 2.23^b^	67.93 ± 3.26^a^
	Therapeutic Silymarin	90.23 ± 3.22^d^	21.18 ± 1.82^f^	42.39 ± 3.64^d^
	Therapeutic *T. lucida*	94.47 ± 5.55^d^	18.54 ± 0.78^d^	31.10 ± 3.30^e^
Protective group	*CCl_4_ Control*	153.08 ± 4.09^a^	42.84 ± 2.65^a^	52.32 ± 4.11^b^
	Protective Silymarin	93.79 ± 3.28^d^	25.57 ± 1.45^e^	32.89 ± 2.90^e^
	Protective *T. lucida*	101.06 ± 5.92^c^	24.89 ± 0.87^e^	34.16 ± 0.84^e^

Data are presented as the means ± S.D of eight replicates. Data analysed by ANOVA one-way, *p* ≤ 0.05, Value with the same letter has no significant but value with different letter has significant at 0.05.

#### The effects of TLRE on histopathological results

**Figure 5.**
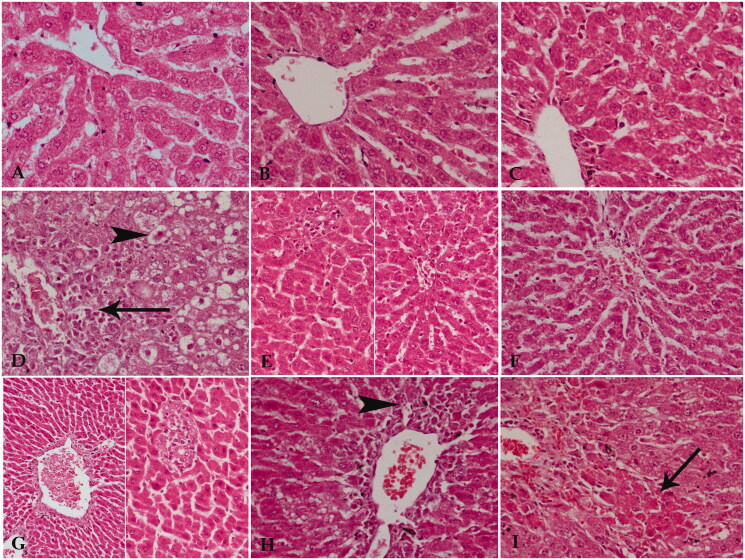
A photomicrograph of liver tissue sections from A) Liver of the negative control rats showed the normal structure of this tissue. B) Livers of positive control of silymarin showed normal structure but with slightly dilated blood sinusoids. C) Livers of positive control of *Tagetes lucida* root extract showed quite normal architecture of liver tissue. D) Livers of therapeutic control showed a significant distortion of the general architecture of the tissue, necrotic cells around the central veins (arrow), and vacuolar degeneration of many cells at the periphery of the lobules (arrowhead). E) Livers of silymarin-treated therapeutic rats showed a marked amelioration of liver tissue except for small focal areas of necrotic cells and/or dilated blood sinusoids. F) Livers of *Tagetes lucida* root extract-treated therapeutic rats showed liver tissue close to normal but with few inflammatory cells around central veins. G) Livers of the protective control rats showed dilatation and congestion of main blood vessels with fibrosis. The parenchyma of liver tissue shows a focal aggregation of necrotic cells. H) Livers of silymarin-treated protective rats showed inflammatory and necrotic cells around dilated central veins (arrowhead). I) Livers of *Tagetes lucida* root extract-treated protective rats showed mild protective effect as areas of acidophilic degenerated cells were observed (arrowhead). Hx & E × 400.

In examining the liver tissue sections, the livers of the negative control group appeared to have normal morphology and histology. The liver tissues for the negative control group appeared grossly brown-red. They presented normal hepatic lobules with normal central veins, hepatic cords, sinusoids, and portal tracts (Figure 5(A)). Using either silymarin or TLRE had no damaging effects on liver tissue (Figure 5(B,C)). Sections in both cases appeared similar to those of the negative control.

On the other hand, the therapeutic control group that first received CCl_4_ and after two days later received saline solution exhibited massive damage to the liver tissue in the form of necrosis and vascular degeneration of cells with distortion of the general architecture of the tissue (Figure 5(D)). On the other hand, silymarin treatment resulted in a marked amelioration of the liver tissue, with small focal areas of necrotic cells (Figure 5(E)). Additionally, TLRE protected the liver tissue from CCl_4_-induced liver damage; the liver tissue appeared similar to the livers of the negative control group (Figure 5(F)). Thus, TLRE gave better results as a therapeutic agent, shifting the tissue towards normal.

Also, the protective control group that received CCl_4_ last after administering saline for one week exhibited damage in the liver. Liver sections from the protective control group showed dilatation and congestion of blood vessels with fibrosis around them. Also, focal areas of necrotic cells were observed in the hepatic lobules of protective control livers (Figure 5(G)). Silymarin had a mild protective effect against the damage from CCl_4_. However, inflammatory and necrotic cells were still observed around the dilated congested blood vessels in the livers of the silymarin protective group (Figure 5(H)). TLRE had a slightly better protective effect than that of silymarin, as reduction of the dilatation and congestion of blood vessels was observed, but acidophilic degenerated cells were also present in the hepatic lobules of the extract-treated groups.

## Discussion

CCl_4_ is a highly hepatotoxic reagent due to its metabolism to trichloromethyl radicals (CCl_3_· and CCl_3_O_2_·) that are generated during cytochrome P-450 action on CCl_4_. These radicals are responsible for lipid peroxidation, inflammation, and fatty changes of the liver. Moreover, CCl_4_ metabolites are linked with oxidative stress. The CCl_3_· and CCl_3_O_2_· radicals disrupted the liver antioxidant system by inhibiting the liver’s antioxidant enzymes as CAT, SOD, GPx, GR, and GST. In addition, the CCl_3_· radicals deactivate GSH via reaction with its SH groups (Andritoiu et al. 2014).

In the present study, administration of CCl_4_ markedly increased liver enzymes; AST, ALT, and ALP, and total bilirubin in the bloodstream coincided with the remarked decrease in protein synthesis and its fractions; albumin and globulin. Such results indicate cellular leakages and loss of functional integrity of cell membranes in the liver due to CCl_4_-mediated hepatotoxicity. The depletion in protein synthesis may be due to the production of aldehydes during the lipid peroxidation process, which blocks the synthesis and secretion of proteins in the serum due to CCl_4_ exposure (Dianzani et al. 1984). CCl_4_-mediated hepatotoxicity was confirmed by histopathological studies, which represented massive damage in the liver tissue. Marked necrosis, distortion of general architecture, vascular degeneration, dilatation, congestion of blood vessels with fibrosis around them, and fatty changes were observed in the liver.

Additionally, the MDA levels in the serum and liver tissue increased markedly due to CCl_4_ administration, synchronised with a significant reduction in GPx, SOD, GST, GSH, and GR in the liver tissue, indicating oxidative damage of the liver. Furthermore, inflammation was taking place, shown by the significant increase in TNF-α associated with CCl_4_ administration. Finally, CCl_4_ administration significantly elevated the CYT2E1 activity that is required for CCl_4_ metabolism.

TLRE significantly decreased the levels of AST, ALT, and ALP enzymes in the bloodstream, moving them towards the normal level, indicating that TLRE protected the structural integrity of the hepatocellular membrane and liver cells against damage caused by CCl_4_. The decrease in elevated bilirubin level and an increase in the TP level and its fractions of albumin and globulin indicated a reduction in the injury to the hepatic parenchyma. Also, TLRE returned the level of MDA to a normal level, indicating an inhibitory effect against lipid peroxidation that prevented the liver injury induced by free radicals along with the subsequent pathological changes in the liver. TLRE showed antioxidant ability represented as an amelioration in a nonenzymatic antioxidant (GSH) concentration and enzymatic antioxidant activities (GPx, SOD, CAT, GST, and GR). The anti-inflammatory effect of TLRE is evident in the remarked modulation in an inflammatory biomarker, TNF-α content. TLRE acted as a CYT2E1 inhibitor and modulated its activity to the normal level.

From previous data, we concluded that TLRE had hepatoprotective features. The hepatoprotective effects of TLRE may be related to its antioxidant aspects, anti-inflammatory features, CYP2E1 inhibition. Also, its content from polyphenols, flavonoids, and alkaloids, and its active constituents gallic acid, chlorogenic acid, naringenin, and quercetin.

Oxidative stress has been implicated in the pathogenesis of various liver diseases, including alcoholic liver disease, non-alcoholic fatty liver disease, and chronic hepatitis C (Saha et al. 2019). By preventing lipid oxidation, antioxidants can prevent CCl_4_ toxicity, especially hepatotoxicity, resulting in decreased levels of released ALT and AST in the bloodstream and activation of an antioxidant enzyme (Xu et al. 2008). SOD is an essential enzyme in the cells of all organisms and can scavenge superoxide radicals. SOD can neutralise superoxide radicals and inhibit the large amounts of released free radicals that CCl_4_ mediates in the early stage (Shah et al. 2015). In the current study, TLRE significantly activated SOD in the case of CCl_4_-induced hepatic damage in rats. GSH plays a crucial role in protecting cells from oxidative injury, reducing H_2_O_2_, hydroperoxides, and xenobiotics. Depletion in hepatic GSH is linked to hepatic injury (Pushpakiran et al. 2004). Therefore, it seems that GSH conjugation is crucially vital for handling CCl_4_-induced hepatic damage. The hepatoprotective action of TLRE may be attributed to several mechanisms including, **a)** activation of antioxidant enzymes (SOD, CAT, GR, GST, and GPx), **b)** protection GSH, **c)** inhibition lipid peroxidation, and **d)** reduction of hydrogen peroxide.

Healthy hepatocytes are insensitive to TNF-α action, but they become sensitive once protein, and RNA synthesis is inhibited. Liver disease or liver failure is associated with the upregulation and downregulation of inflammatory biomarkers. TNF-α and NO have a vital function in inducing inflammation in oxidative stress-related hepatic diseases, promoting programmed cell death and fibrosis. TNF-α is an important cytokine that promotes hepatic damage (Ahmed et al. 2011). Nayeli et al. (2018**)** reported the anti-inflammatory activity of TLRE coumarin in a mouse model of 12-*Ο*-tetradecanoylphorbol 13-acetate (TPA)-induced auricular edoema.

CCl_4_ is metabolised by CYP2E1, releasing free radicals, which injure the liver cell. In addition, CCl_4_ metabolites interact with the unsaturated fatty acids of cell membranes, causing lipid peroxidation (Takahashi et al. 2002). The current study demonstrates that CCl_4_ promotes CYP2E1 activity while TLRE inhibits it, where TLRE protected lipids from peroxidation.

TLRE contains many chemical compounds that have hepatoprotective, anti-inflammatory, antioxidant and CYP2E1-inhibitory effects. These compounds are including polyphenols (as gallic acid, chlorogenic acid, syringic acid, ellagic acid, ferulic acid, and cinnamic acid), flavonoids (as naringenin and quercetin), alkaloids [as corynan-17-ol, 18,19-didehydro-10-methoxy-, acetate (ester) component], and coumarins (as coumaric acid).

Phenolic compounds downregulate liver enzyme biomarkers; AST and ALT. They can also restrain the oxidative stress and activation of T cells, a prime instigator of inflammation (Saha et al. 2019). In addition, phenolic compounds offer protection against oxidative damages by donating hydrogen or electrons to free radicals. Thus, in this process, they aid in stabilising cell membrane networks and inhibiting the formation and expression of inflammatory cytokines like TNF-α (Saha et al. 2019). TLRE contains polyphenols at 12.62 ± 1.21 mg gallic acid/g extract.

Gallic acid exhibits hepatoprotective activity against CCl_4_ mediated liver fibrosis (Sun et al. 2014) via activation of the hepatic stellate cell (HSC), reduction in oxidative stress biomarker; MDA, elevation SOD, and ATPases (Ca^2+^/Mg^2+^), attenuating the inflammatory mediators COX-2 through NF-kB inhibition pathway. TLRE contains gallic acid as a 399.76 μg/g extract, which may help in liver protection. Chlorogenic acid has a hepatoprotective effect in CCl_4_-induced hepatotoxicity rats via free radical-scavenging and antioxidative activity. Chromogenic acid (1426.04 μg) occurs in each gram of TLRE. Quercetin and ellagic acid have potent hepatoprotective, antioxidant, and anti-inflammatory activities against hepatotoxicity. Administration of QR and EA showed hepatoprotective effect through reducing liver biomarkers, improving the redox status of the tissue, and hampering the expression level of fibrosis-related genes (Afifi et al. 2018). Each gram of TLRE contains 180.25 μg ellagic acid and 4182.24 μg quercetin, responsible for the hepatoprotective effect in the current study. *p*-Coumaric acid exhibits hepatoprotective via lipid peroxidation inhibition. It prevents the accumulation of malondialdehyde and decreases SOD activity and GSH levels (Akdemir et al. 2017). TLRE contains *p*-coumaric acid by about 58.74 µg/g. Ferulic acid has the potent antioxidant ability to freeze the activity of free radicals like NO, O**^−^**^2^ (Cheng 2018). In addition, it has anticholestatic effect against liver cholestasis. Also, it can reduce lipid oxidation that is essential in treating fatty liver (Saha et al. 2019). In this study, TLRE modulated the lipid metabolism due to ferulic acid (208.22 μg/g extract).

In addition, cinnamic acid has a hepatoprotective action against cisplatin-induced hepatotoxicity in mice (Tohamy et al. 2016). It mediated hepatotoxicity via decreasing oxidative stress biomarkers (MDA and NO) and increasing GSH concentration. Also, it has anti-inflammatory effects as a reduction in NF-κB activity resulting in a significant increase in NO production (Cichocki et al. 2010). TLRE exhibited anti-inflammatory action may result due to cinnamic acid (408.94 μg/g extract). Itoh et al., 2010 reported that syringic acid and vanillic acid could suppress hepatic fibrosis in CCl_4_-induced chronic liver injury. They prevented lipid peroxidation, decreased liver hydroxyl proline content, and improved liver histology. TLRE contains 143.80 µg syringic acid/g extract and 198.18 µg vanillin/g extract.

Flavonoids have several biological activities, including hepatoprotective, anti-inflammatory, antidiabetic, antihyperlipidemic, and antiviral activities. Flavonoids prevent oxidative stress by directly scavenging free radicals, metal chelation, reduction capacity, induction of antioxidant enzymes, and phase II detoxifying enzymes such as GST. Flavonoids also have a membrane-stabilising effect. Flavonoids can interact with CYP450 enzymes by modulating their biosynthesis (Östlund 2017). Quercetin inhibits CYP2E1 activity. Also, quercetin has a protective effect against CCl_4_-induced liver damage in mice via enhancing the antioxidative defense system and suppressing the inflammatory response (Östlund 2017). CYP2E1 modulation may be attributed to the quercetin content in the extract, where each gram of TLRE contains 4182.24 μg quercetin. Additionally, the hepatoprotective of TLRE may associate with its content of flavonoids that act about 5.64 ± 0.44 mg quercetin/g. Naringenin exhibits hepatoprotective activity due to the normalisation of the liver functions, suppression of oxidative stress, activation of antioxidant enzymes, and preventing fibrosis formation (Afifi et al. 2018). TLRE contains a high content of naringenin; 1419.65 μg/g extract.

Many studies have demonstrated the hepatoprotective characteristics of alkaloids in many plants. Alkaloids have good hepatoprotective activity by inhibiting MDA and activating antioxidant enzyme activities (Sangale and Patil 2017). TLRE in this study was rich in active alkaloids, at 300.85 ± 10.25 mg/g extract.

The remarkable hepatoprotective, anti-inflammatory, and antioxidant effect of TLRE may attribute to a synergistic effect between the mentioned compounds.

## Conclusions

In this study, tagetes extract exhibited a hepatoprotective effect against CCl_4_-induced hepatotoxicity rats. Liver histology and performance of CCl_4_-induced hepatotoxicity rats were returned towards normal levels. TLRE suppressed the activity of CYP2E1 and modulated the synthesis of TNF-α. Additionally, TLRE upregulated antioxidant biomarkers and downregulated oxidative stress biomarkers. These support the conclusion that the promising hepatoprotective activity of TLRE may be due to its antioxidant and anti-inflammatory characteristics and CYP2E1-inhibitory effect, which may, in turn, be attributed to its content of polyphenols, flavonoids, coumarins, and crude alkaloids. This study can be generalised to a broader study population and clinical trial as the extract we studied is completely safe. Future research can evaluate the effect (protective and/or therapeutic) on other organs.
